# Multidisciplinary treatment incorporating autologous DC-CIK cell therapy induces long-term survival in advanced pancreatic cancer: a case report

**DOI:** 10.3389/fimmu.2026.1908017

**Published:** 2026-09-18

**Authors:** Shanshan Chen, Yunqing Xie, Shaohua Xu, Lili Su, Yangmei Xu, Shijia Liu, Qiuhong Zheng, Qinying Liu, Yang Sun

**Affiliations:** 1 Fujian Provincial Key Laboratory of Tumor Biotherapy, Clinical Oncology School of Fujian Medical University, Fujian Cancer Hospital, Fuzhou, China; 2 Department of Hepatobiliary and Pancreatic Surgery, Clinical Oncology School of Fujian Medical University, Fujian Cancer Hospital, Fuzhou, China; 3 Cell Therapy Research Center, Xiamen Humanity Hospital, Xiamen, China; 4 Department of Gynecology, Clinical Oncology School of Fujian Medical University, Fujian Cancer Hospital, Fuzhou, China

**Keywords:** advanced pancreatic cancer, case report, multidisciplinary treatment, DC-CIK cells, long-term survival

## Abstract

Pancreatic cancer is widely acknowledged as one of the most aggressive malignancies of the digestive system. More than 80% of patients are diagnosed at an advanced stage with an extremely poor prognosis. While contemporary combination chemotherapy and the novel RAS inhibitor daraxonrasib have modestly prolonged survival, clinical benefits remain limited, necessitating less toxic yet more substantial and durable therapeutic strategies. Herein, we report a case of a patient with advanced metastatic adenocarcinoma who achieved disease stability and long-term survival following multidisciplinary treatment incorporating autologous DC-CIK cell therapy. The patient was a 73-year-old male diagnosed with poorly differentiated pancreatic ductal carcinoma 9 years ago. After surgery, he developed pelvic implantation metastases and subsequently received multi-line chemotherapy and radiotherapy. Thereafter, he completed five cycles of DC-CIK cell infusion at a dose of 5×10^9^ ~10×10^9^ cells between November 2018 and December 2019. The patient did not experience any obvious adverse reactions during the treatment. Post-treatment, the patient maintained a stable disease state. Although bone metastasis was detected in February 2021, the patient’s overall survival (OS) and progression-free survival (PFS) reached 107 and 49 months, respectively, far exceeding those previously reported in the literature. Furthermore, the patient experienced an improvement in quality of life. These findings support multidisciplinary care as the cornerstone of long-term survival in pancreatic cancer. As an integral component of immunomodulation, DC-CIK cell therapy remodels the tumor microenvironment to facilitate other treatments, making it a defining therapeutic decision for patients with advanced pancreatic cancer.

## 1 Introduction

Pancreatic cancer (PC) remains one of the most lethal malignancies worldwide. According to the American Cancer Society’s Cancer Facts & Figure 2025, the 5-year relative survival rate is only 13%^1^. Pancreatic cancer is characterized by an insidious onset and rapid progression; over 80% of patients present with locally advanced or metastatic disease that precludes curative-intent surgery (1).

Although modern combination chemotherapy—principally FOLFIRINOX (fluorouracil, leucovorin, irinotecan, and oxaliplatin) and NALIRIFOX (fluorouracil, leucovorin, liposomal irinotecan and oxaliplatin)—has extended median OS in metastatic PC to 10.4 to 11.7 months, incremental gains have plateaued and treatment-related toxicity remains substantial (2, 3). Thus, novel, less toxic strategies are urgently needed. The novel therapeutic approaches including immune-checkpoint inhibitors, CAR-T/TCR-T cells and therapeutic vaccines, have preliminarily shown antitumor activity in pancreatic cancer (4). However, due to bottlenecks such as a profoundly immunosuppressive tumor microenvironment, scarce T-cell infiltration, and potential off-target toxicities, single-agent efficacy remains low and these approaches is still in the clinical exploration stage (5–10).

Non-genetically modified adoptive cell therapies (ACTs)—such as dendritic cell–cytokine-induced killer (DC-CIK) cells, tumor-infiltrating lymphocytes (TILs), and natural killer (NK) cells—aim to reconstitute antitumor immunity through the ex vivo activation and expansion of autologous immune cells prior to reinfusion. DC-CIK cells, generated by co-culturing CIK cells with antigen-loaded DCs, combine the MHC-unrestricted cytotoxicity of CIK cells with the potent antigen-presenting capacity of DCs (11, 12). They have been proven to prolong survival, improve quality of life (QoL), and cause minimal toxic side effects in solid tumors, offering a promising option for advanced pancreatic cancer (12–14).

Mechanistic investigations indicate that DC-CIK infusion significantly increases peripheral CD3^+^, CD3^-^CD56^+^, CD3^+^CD56^+^, and CD4^+^/CD8^+^ ratios, and promotes the release of antitumor cytokines such as IFN-γ, IL-2, and TNF-α while reducing CA19–9 and CEA levels, consistent with the restoration of systemic antitumor immunity (12, 13, 15). Although large randomized trials are lacking, case series report that multiple DC-CIK infusions have achieved PFS exceeding 19 months in patient intolerant of conventional therapy, together with marked QoL improvement (16). These findings provide clinical evidence that cell therapy promotes long-term survival in cancer patients.

Here, we describe a patient with metastatic pancreatic cancer who achieved an exceptional OS of 107 months and PFS of 49 months after multidisciplinary treatment incorporating autologous DC-CIK cell therapy, with sustained QoL. DC-CIK treatment expanded cytotoxic immune cell subsets, greatly improved life quality and displayed satisfactory safety without severe adverse events. Written informed consent was obtained for publication.

## 2 Case description

The patient, a 73-year-old male, was admitted to our hospital in January 2017 with a chief complaint of “progressive yellowing of the eyes for more than 1 months”, accompanied by yellow discoloration of the skin, dark urine, generalized pruritus, and clay-colored stools. Physical examination revealed slight icteric sclerae and tympanic percussion note over the abdomen. Endoscopic Retrograde Cholangiopancreatography (ERCP) demonstrated stenosis of the lower common bile duct, with suspicion of cholangiocarcinoma or pancreatic carcinoma infiltration. Biopsy histopathological examination of the lower common bile duct revealed biliary epithelium with moderately to poorly differentiated adenocarcinoma in the stroma. On January 5, 2017, he underwent pancreaticoduodenectomy with partial portal vein resection and reconstruction. Intraoperatively, a firm, 2.5 cm mass was palpated in the pancreatic head and was adherent to surrounding tissues. The pancreatic body and tail appeared small and atrophic, and no palpable mass was identified. The abdominal cavity was free of ascites. No seeding nodules were detected in the abdominal wall or pelvic floor. Postoperative histopathology revealed: (a) Pancreaticoduodenectomy specimen: poorly differentiated ductal adenocarcinoma of the pancreatic head extending beyond the pancreatic capsule, with invasion of the common bile duct, ampulla of Vater, duodenal wall, perineural spaces and vascular–lymphatic channels. Malignant cells infiltrate the pancreatic transection margin with tumor thrombi identified within vascular-lymphatic channels. (b) Portal vein wall: adenocarcinoma cells infiltrating the vascular smooth-muscle layer. Immunohistochemistry results are summarized below: Ki67(40%+), CK7 (++), CK20 (-), villin (+), CEA (+++), CDX-2 (+), CD56 (-), CD34 (-).

Postoperative PET-CT indicated pelvic implantation. Therefore, six cycles of FOLFIRINOX chemotherapy regimen were administered from March 1 to May 28, 2017, along with 5 intraperitoneal (ip) infusions of FUDR and 1 ip infusion of Endostar. The total dosages used were as follows: CPT-11 (Irinotecan): 1280 mg; OXA (Oxaliplatin): 1000 mg; L-CF (Leucovorin): 1.8 g; FUDR (Floxuridine): 5.0 g; 5-FU (Fluorouracil): 13.0 g; Endostar: 60 mg. Tumor response after three cycles of chemotherapy was stable disease (SD). From July 5 to August 16, 2017, the patient received intensity-modulated radiotherapy (IMRT) at Fujian Cancer Hospital. Clinical target volume (CTV) included the preoperative tumor area and the portal vein involved as shown by postoperative pathology. Planning target volume (PTV): CTV plus a 5–10 mm margin. 95% TV received 5600 cGy in 28 fractions. 95% of GTV-P received 30 Gy in 10 fractions. During radiotherapy, grade 3 bone marrow suppression occurred and improved after symptomatic treatment. Pelvic IMRT was performed from September 7 to September 20, 2017. From October 13 to October 30, 2017, and from January 5 to January 9, 2018, two cycles of capecitabine (1.0 g orally, twice daily, days 1–14) were administered.

The patient began receiving DC-CIK cell therapy in November 2018, with approximately one treatment course every two months. A total of five cycles were completed between November 2018 and December 2019. The patient tolerated the treatment well, with no significant adverse events observed during the treatment period.

Throughout DC-CIK therapy and subsequent follow-up, the patient underwent regular monitoring of pancreatic tumor markers CA19-9, CEA, and AFP as well as peripheral blood counts (**Figures 1A, B**).

**Figure 1:**
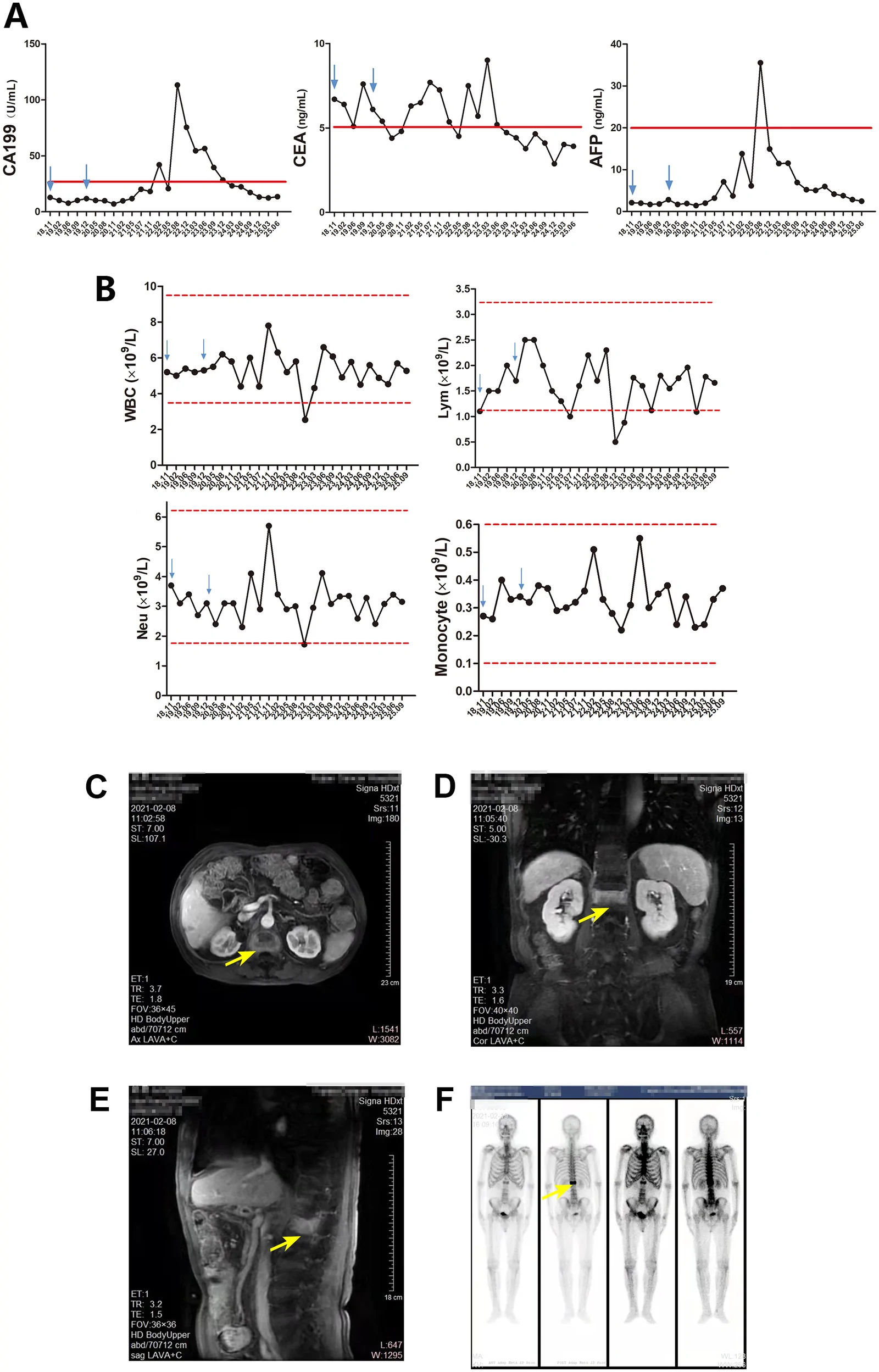
**(A, B)** Dynamic changes in expression levels of tumor biomarkers (CA199, CEA and AFP) and leukocyte subpopulations in peripheral blood from 2018.11 to 2025.09. **(A)** Changes in the expression levels of CA199 (left), CEA (middle) and AFP (right); **(B)** Dynamic changes in WBC, lymphocytes, neutrophils, and monocytes. MRI (C, D&E) and ECT **(F)** findings after bone metastasis to the L1 vertebral body. (Blue arrow, DC-CIK treatment period; Yellow arrow, L1 vertebral body metastasis; CA199, Cancer Antigen 19-9; CEA, Carcinoembryonic Antigen; AFP, Alpha-fetoprotein; WBC, White Blood Cells; MRI, Magnetic Resonance Imaging; ECT, Emission Computed Tomography.).

On February 8, 2021, follow-up abdominal CT revealed an abnormal signal in the L1 vertebral body, raising concern for metastasis; ECT confirmed focally increased radionuclide uptake at L1, consistent with osseous metastasis (**Figures 1C–F**). Intensity-modulated radiotherapy to the lumbar spine was delivered from March 9 to March 15, 2021. The L1 vertebral body was designated as CTV, expanded by 5 mm to create the PTV, and the 95% of PTV receiving 3–000 cGy in 5 fractions.

Abdominal MRI on October 15, 2022 revealed enlargement of the L1 and L2 vertebral metastases, prompting re-irradiation from November 16 to December 5, 2022, during which L1 and L2 were designated CTV, a 5 mm margin formed the PTV, the PTV first received 1.5 Gy twice daily for 10 days and then a single 500 cGy boost, followed by monthly zoledronic acid to inhibit osteoclast-mediated bone destruction and prevent hypercalcaemia.

Lumbar CT on April 26, 2023 demonstrated stable L1 and L2 lesions, yielding a response of stable disease, and regular denosumab was continued with the last injection on September 11, 2025. On August 28, 2025, the patient presented to hospital with one month of low-back pain and restricted mobility; lumbar MRI showed a compression fracture of the T12 vertebral body with bone-marrow oedema. After surgical contraindications were excluded, percutaneous vertebroplasty of T12 was performed under local anaesthesia on September 1, 2025, and post-operative thoracolumbar radiographs confirmed vertebroplasty changes at T12; post-cement-injection changes of pathological fractures at L1–L2, marked height loss of L1–L2, and degenerative lumbar changes.

Medical, family, and psycho-social history: Hypertension for 18 years, treated with amlodipine 5 mg once daily, metoprolol 25 mg once daily, and aspirin 100 mg once daily. Type 2 diabetes mellitus for 11 years, on insulin therapy. Status post partial right thyroidectomy for hyperthyroidism performed over 30 years ago; currently euthyroid. No family history of malignancy, genetic disorders, or endocrine diseases was noted. No history of smoking or alcohol consumption.

The current diagnosis of this patient: 1) Poorly differentiated pancreatic-head adenocarcinoma involving the distal common bile duct, ampulla of Vater and duodenum (pT3N0M0 stage II), peritoneal, pelvic seeding and bone metastasis post resection (rT3N0M1 stage IV), following multiple lines of treatment; 2) Grade 3 hypertension (very high risk);3) type 2 diabetes mellitus (T2DM);4) status post partial right thyroidectomy.

As of December 2025, the patient has achieved a PFS of 49 months and an OS of 107 months (8 years and 11 months). (**Figure 2**).

**Figure 2:**
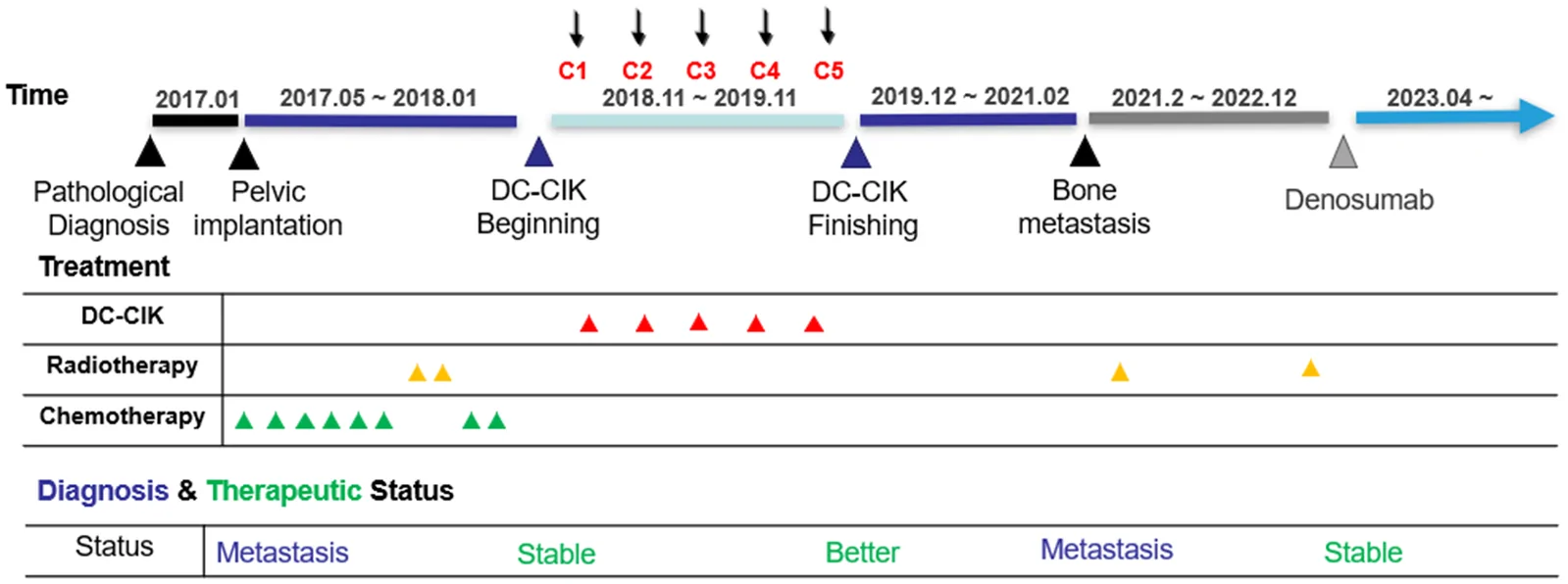
Treatment process and therapeutic outcomes from 2017.1 to 2025.12 (the timeline).

## 3 Methods

### 3.1 DC-CIK cells manufacture

Before each cycle, 50 mL of peripheral venous blood was collected. Peripheral blood mononuclear cells (PBMCs) were isolated using Ficoll (GE Healthcare Life Sciences (Chalfont, UK) density gradient centrifugation. Peripheral blood was diluted 1:1 with PBS and carefully overlaid onto an equal volume of Ficoll-Paque (1.077 g/mL). Following centrifugation at 300×g for 25 min at room temperature with minimal acceleration and deceleration (both set to 1), the PBMC layer at the interface was collected, washed twice with PBS (200×g, 20 min), and counted by trypan blue staining. The PBMCs were then cultured in serum-free medium for 4–6 hours to allow adherence. Adherent cells were used for dendritic cell (DC) culture. Suspension cells were used for cytokine-induced killer (CIK) cell culture. DCs were stimulated with GM-CSF (60ng/mL) (Sino Biological, Beijing, China) and IL-4 (75ng/mL) (Sino Biological), while CIK cells were stimulated with anti-CD3 monoclonal antibody (10μg/mL) (R&D Systems, USA), IL-2 (500U/mL) (Sino Biological), and IFN-γ (500U/mL) (Sino Biological). Additionally, on day 5 of culture, DCs were pulsed with WT1 protein (75 ng/mL) (Miltenyi Biotec, Bergisch Gladbach, Germany) to facilitate antigen loading and maturation. On day 8, mature DCs were co-cultured with CIK cells (**Figures 3A, B**). On days 11–12, following completion of pathogen screening and confirmation of immune-cell viability and phenotype (**Figures 3D–F**), the cells were administered to the patient via intravenous infusion.

**Figure 3:**
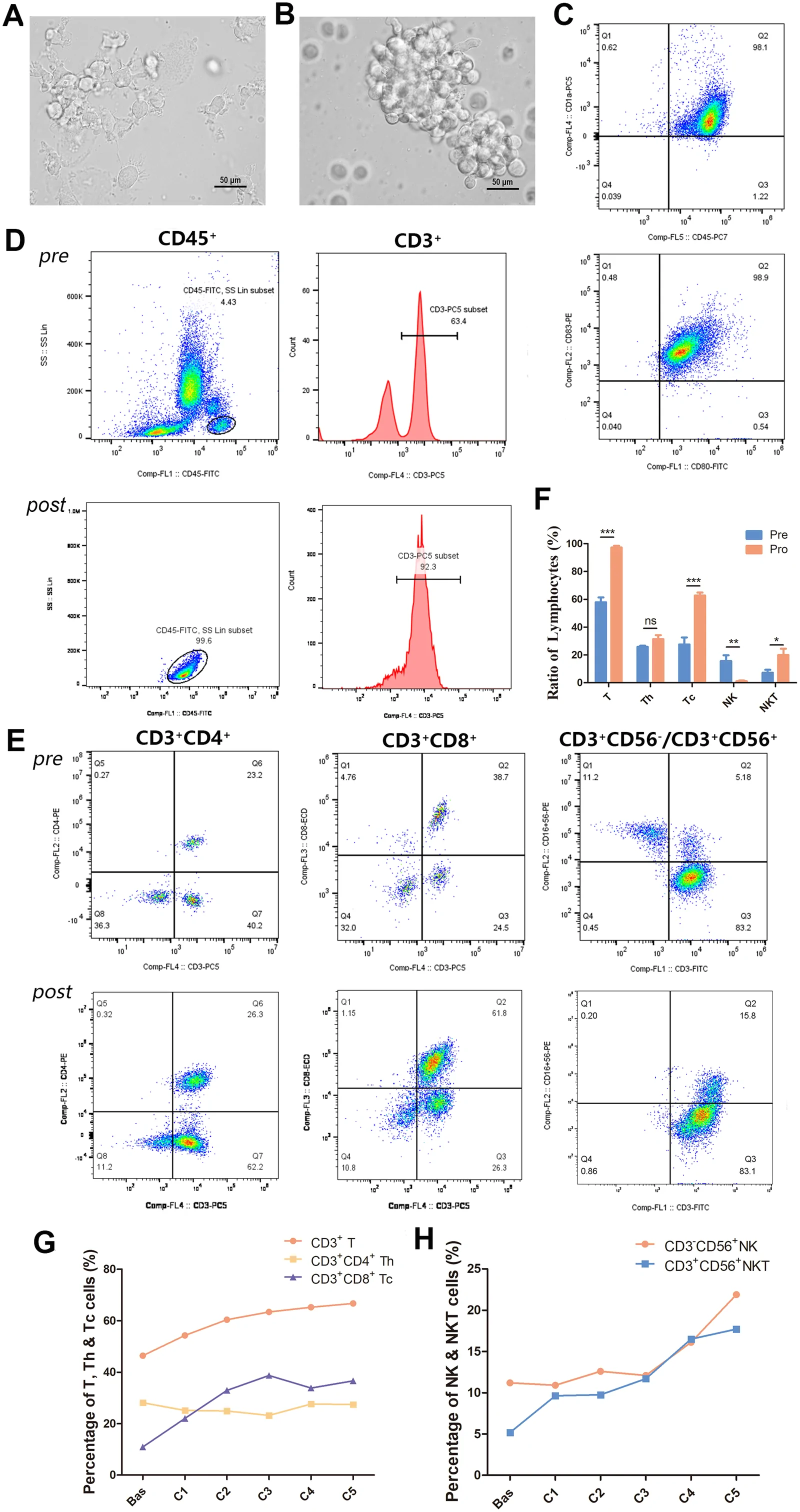
Representative microscopic images of cultured DC **(A)** and CIK **(B)** cells, and flow cytometry dot plots of mature DC cells **(C)**. Changes in T cell and NK cell subsets before and after DC-CIK cell culture **(D–F)**. **(D)** Changes in CD45^+^ lymphocyte (left) and CD3^+^ T(right) cell proportions before and after culture. **(E)** Alterations in the percentages of CD3^+^CD4^+^ helper T cells (Th) (left), CD3^+^CD8^+^ cytotoxic T cells (Tc) (middle) and CD3^+^CD56^+^ NKT like cells (right) Pre- and Post-Culture. **(F)** Bar chart showing the relative proportions of immune cell subsets before and after *in vitro* culture (Data are presented as mean ± SEM, *P<0.05, **P<0.01, ***P<0.001). Dynamic changes in T **(G)**, NK and NKT **(H)** cell subsets in peripheral blood during DC-CIK cell therapy. (Bas: baseline; C1~C5: Cycle 1~5).

All products tested negative for bacteria, fungi, and mycoplasma, with endotoxin levels below 5 EU. The number of DC-CIK cells infused per dose ranged from 5×10^9^ to 10×10^9^.

### 3.2 Flow cytometry

To identify the phenotypes of cultured DCs and CIK cells, flow cytometric analysis was performed. The following antibodies were used for flow cytometry: CD45-FITC, CD4-RD1, CD8-ECD, CD3-PC5 (cat. no. 6607013), CD3-FITC, CD (16 + 56)-PE (cat. no. A07735). CD45-PC7(cat. no.IM3548), CD1a-PC5 (cat. no. IM3610), CD80-FITC (cat. no. IM1853U), CD83-PE (cat. no. IM2218U) (All from Beckman Coulter, Inc.; Brea, California, USA).

Cells were harvested, centrifuged at 200×g for 5 min, and the supernatant was discarded. The cells were then resuspended in 100 μL of PBS, and 5 μL of detection antibodies were added. After incubation at room temperature for 20 min protected from light, the cells were washed twice with PBS, resuspended in 300 μL of PBS, and subjected to flow cytometric analysis.

Peripheral blood and cultured T lymphocyte were stained and acquired using two separate antibody panels. *Panel A:* Lymphocytes were first identified based on CD45 expression and side scatter (SSC) properties (CD45/SSC gating). Within the lymphocyte gate, total T cells were identified as CD3-positive (CD3^+^) cells, helper T cells (Th cells) were defined as CD3^+^CD4^+^ cells, and cytotoxic T cells (Tc cells) were defined as CD3^+^CD8^+^ cells. *Panel B:* Lymphocytes were initially gated using CD45/SSC gating) as described above. Within the lymphocyte gate, NK cells were identified as CD3^+^(CD16 + 56)^+^ cells, and NKT cells were identified as CD3^+^(CD16 + 56)^-^ cells. For cultured DC cells, DC cells were first gated as CD45^+^CD1a^+^ cells. Mature DC cells were subsequently identified as CD80^+^CD83^+^ cells within the DC cell population.

Flow cytometric data were acquired on the FC500 flow cytometer (Beckman Coulter, Brea, CA, USA) and analyzed using FlowJo software (version 10.8.1) (Tree Star, Ashland, OR, USA).

After culture, the proportions of CD3^+^ total T cells (P<0.001), CD3^+^CD8^+^ cytotoxic T cells (P<0.001), CD3^+^CD56^+^ NKT like cells (P<0.05) were all significantly higher than before culture (**Figures 3D–F**). In addition, the purity of cultured DC cells (CD45^+^CD1a^+^) exceeded 95%, and their maturation status (CD80^+^CD83^+^) also exceeded 95% (**Figure 3C**).

## 4 Discussion

Long-term survival in advanced pancreatic ductal adenocarcinoma (PDAC) remains an uncommon outcome. Despite the recent breakthrough reported at the 2026 ASCO Annual Meeting—wherein the phase 3 RASolute 302 trial demonstrated that daraxonrasib, an oral RAS(ON) multiselective inhibitor, nearly doubled median overall survival to 13.2 months compared with 6.7 months for standard chemotherapy in previously treated metastatic PDAC (17, 18)—these data underscore that even highly effective targeted monotherapies achieve only modest absolute survival benefits (Compared with the previously described combination chemotherapy that yielded a maximum survival of 11.7 months) (2) in this disease. Moreover, historical data indicate that the 5-year survival rate for all stages of PDAC not exceeding 13% even in the modern era.^1^.

In fact, prolonged survival in pancreatic cancer is rarely attributable to a single intervention. Durable disease control appears to emerge from the systematic integration of multiple treatment pillars—surgical resection, systemic chemotherapy, locoregional radiotherapy, and emerging immunotherapeutic strategies—coordinated within a multidisciplinary framework.

In the present case, the patient developed pelvic metastases after surgery—a pattern consistent with the aggressive biological behavior of this malignancy. Following high-dose chemoradiotherapy, he experienced anorexia, fatigue, and asthenia. With the informed consent of both the patient and his family, we initiated DC-CIK cellular immunotherapy. After five cycles, his EORTC QLQ-C30 (European Organisation for Research and Treatment of Cancer Quality of Life Questionnaire-Core 30) score rose from 67 to 83, nausea/vomiting and pain subsided, appetite returned to normal, and overall quality of life was markedly improved (**Table 1**).

**Table 1:** Changes in the patient’s quality of life following DC-CIK cell therapy.

Scales	Pre-treatment	Post-treatment				
		1 cycle	2 cycles	3 cycles	4 cycles	5 cycles
Function subscales						
Physical function	80	80	80	80	87	87
Role function	67	83	83	83	83	83
Emotional function	75	83	83	83	83	92
Cognitive function	83	83	83	83	83	83
Social function	100	100	100	100	100	100
Symtom Scales & Single items						
Fatigue	33	33	33	33	33	33
Nausea / Vomiting	17	0	0	0	0	0
Pain	33	33	33	33	17	17
Dyspnea	33	0	0	0	0	0
Insomnia	33	0	0	0	0	0
Appetite loss	33	33	33	0	0	0
Constipation	33	33	33	33	33	33
Diarrhea	0	0	0	0	0	0
Financial difficulties	0	0	0	0	0	0
Global health / Quality of life	67	67	75	75	75	83

Bold values represent the four major dimension categories of the EORTC QLQ-C30.

Although bone metastases emerged one year after cellular therapy was discontinued, the patient’s OS and PFS have already exceeded the median values reported for advanced pancreatic cancer (2). Throughout the course of DC-CIK cell therapy, serum tumor markers (CEA, CA19-9, AFP) and peripheral-blood counts of WBC, lymphocytes, neutrophils, and monocytes remained within or close to the normal ranges (**Figures 1A, B**). Concurrently, immunophenotyping showed a progressive rise in the frequencies of CD3^+^ total T cells, CD8^+^ cytotoxic T cells, CD3⁻CD56^+^ NK cells, and CD3^+^CD56^+^ NKT cells (**Figures 3G, H**).

In recent years, adoptive cellular immunotherapy represented by DC-CIK has developed rapidly in the field of solid tumor treatment. Dendritic cells are the most potent professional antigen-presenting cells in the body, while CIK cells are a heterogeneous population of T lymphocytes activated by various cytokines, among which CD3^+^CD56^+^ T cells are the main effector cells (19, 20). Co-expanding tumor-antigen-loaded DCs with CIK ex vivo generates a synergistic, robust, and durable antitumor immune response (12).

The synergistic antitumor action of DC-CIK rests on three pillars: (a) DCs capture, process, and present tumor antigens to CIK cells, driving their maturation and stimulating their release of cytokines such as IL-2, IFN-γ, and TNF-α to suppress tumor growth and boost systemic antitumor immunity. (b) Once activated, CIK cells discharge perforin- and granzyme-bearing cytotoxic granules that directly puncture and lyse tumor cells. (c) The engagement of the Fas/FasL pathway ignites apoptotic cascades within the malignant population, completing the triad of tumor destruction (11, 19, 21, 22).

Unlike the ephemeral cell-killing effects of conventional chemotherapy, DC-CIK immunotherapy is characterized by a prolonged duration of action and prominent long-term therapeutic benefits. Case reports have demonstrated that advanced pancreatic cancer patients who were unable to tolerate chemoradiotherapy achieved a progression-free survival exceeding 19 months with CIK monotherapy alone, accompanied by normalization of tumor marker (23). Additionally, a phase II study indicated that even in advanced patients with gemcitabine-resistant disease, CIK treatment could still yield a median overall survival of 26.6 weeks while improving quality of life (24).

As a late-stage pancreatic-cancer patient with multiple post-operative metastases for whom conventional options were scarce, this case received DC-CIK cellular therapy in addition to standard chemoradiation. The administration of systemic chemotherapy and locoregional radiotherapy achieved meaningful tumor cytoreduction and disease stabilization. This reduction in tumor burden, coupled with the immunomodulatory consequences of cytotoxic therapy, may have created a favorable therapeutic window for adjunctive immunotherapy. It was against this backdrop of controlled disease that autologous DC-CIK cell therapy was introduced, not as a standalone intervention, but as an immunomodulatory component embedded within a comprehensive, response-adapted treatment strategy.

The intervention not only improved the patient’s quality of life but also slowed the growth of osseous lesions, enabling long-term survival for this patient. At the latest follow-up, OS has reached 107 months, and PFS after the detection of pelvic implants stands at 49 months—offering a promising therapeutic strategy for patients with metastatic pancreatic cancer.

Given the extremely poor prognosis of metastatic pancreatic cancer^1^, and the propensity of chemotherapy to develop primary or secondary resistance, making long-term disease control difficult to achieve (25), the survival outcomes observed in this case substantially surpassed the historical benchmarks of chemotherapy alone, likely attributable to the cytoreductive and microenvironment-modulating groundwork established by conventional therapies, and sustained immunological effects induced by DC-CIK cell therapy.

## 5 Conclusion

In summary, this report presents a patient with advanced pancreatic cancer who achieved markedly prolonged OS and PFS following multidisciplinary treatment incorporating autologous DC-CIK cell therapy, outcomes that substantially surpass those previously reported in the literature (2). This should be interpreted as the cumulative result of integrated multidisciplinary care rather than the isolated effect of any single intervention.

The treatment trajector—encompassing surgical resection, systemic chemotherapy, locoregional radiotherapy, autologous DC-CIK cell immunotherapy, and subsequent metastasis-directed therapy with rigorous supportive care—aligns with contemporary principles of oncologic management, wherein each modality addresses distinct aspects of tumor biology and host resilience. With this framework, DC-CIK cell therapy likely functioned as a valuable immunomodulatory adjunct, potentially enhancing immune surveillance and improving host immunological fitness during periods of minimal residual disease. Notably, the treatment was well tolerated, with no adverse events requiring clinical intervention, and was associated with a meaningful improvement in quality of life. Although bone metastasis ultimately emerged, it occurred more than one year after discontinuation of DC-CIK therapy, implying that durable immune regulation in advanced pancreatic cancer may necessitate prolonged, uninterrupted treatment.

These observations highlight the therapeutic potential of DC-CIK cell therapy for advanced PC, however, as a single-case report, it does not permit disentanglement of the relative contributions of tumor biology, conventional treatments, and cellular immunotherapy. Future prospective investigations, ideally conducted within standardized multimodal protocols with correlative immune monitoring, are warranted to clarify whether DC-CIK cell therapy provides incremental benefit when layered upon optimal conventional therapy.

## Footnotes

1 Available at: https://www.cancer.org/research/cancer-facts-statistics/all-cancer-facts-figures/2025-cancer-facts-figures.html.
